# A Novel Irrigation System to Reduce Heat Generation during Guided Implantology: An In Vitro Study

**DOI:** 10.3390/jcm12123944

**Published:** 2023-06-09

**Authors:** Somayeh Parvizi, Andrew Cameron, Santosh Tadakamadla, Carlos Marcelo S. Figueredo, Peter Reher

**Affiliations:** 1 School of Medicine & Dentistry, Griffith University, Brisbane, QLD 4111, Australia; somayeh.parvizi@griffithuni.edu.au (S.P.); a.cameron@griffith.edu.au (A.C.); p.reher@griffith.edu.au (P.R.); 2 Dentistry and Oral Health, Department of Rural Clinical Sciences, La Trobe Rural Health School, Bendigo, VIC 3552, Australia; s.tadakamadla@latrobe.edu.au; 3 Division of Oral Diseases, Department of Dental Medicine, Karolinska Institutet, OF Odontologi, OF Orala sjukdomar, 171 77 Stockholm, Sweden

**Keywords:** dental implants, surgical guide, irrigation channels, thermocouples, 3D printing, CAD/CAM

## Abstract

The purpose of this in vitro study is to evaluate the effectiveness of incorporating a new irrigation system into a surgical guide and monitor its effect on heat generation during implant bed preparation. A total of 48 surgically guided osteotomies were performed on 12 bovine ribs divided into 4 groups, using different irrigation techniques: Group A (test) had entry and exit channels incorporated into the guide; Group B had a similar design with an entry channel only; Group C had conventional external irrigation; and Group D (control) had no irrigation. Heat generation during the osteotomies was measured using thermocouples placed at a depth of 2 mm and 6 mm. The lowest mean temperature was observed in Group A (22.1 °C at 2 mm and 21.4 °C at 6 mm), which was statistically significant when compared with Groups C and D (*p* < 0.001). Group A showed a lower mean temperature compared with Group B as well; however, it was statistically significant only at 6 mm depth (*p* < 0.05). In conclusion, the proposed surgical guide has significantly reduced heat generation during implant osteotomy compared to conventional external irrigation. The integration of an exit cooling channel can resolve limitations found in previously designed surgical guides such as debris blockage and can be easily incorporated into computer designing and 3D printing software.

## 1. Introduction

Surgical guides are important tools to aid in the insertion of dental implants in the prosthetically driven, digitally planned ideal position with a high level of accuracy. Moreover, regional anatomy is visualized on the implant designing software before surgery and the possibility of iatrogenic injury to vital structures can be minimized [1]. However, 3D-printed guides act as a barrier surrounding implant bed preparation and consequently will minimize or block irrigation fluid from reaching the osteotomy site adequately. Inadequate irrigation during implant drilling can excessively increase the temperature and damage those vital cells crucial for bone remodeling and osteointegration [2,3,4].

Histological evaluation of implant failures stated bone overheating as the most plausible cause of implant failure [5]. Although there is no clear threshold value for heat-induced osteonecrosis available in the literature, most related articles have suggested that during bone drilling temperature should not exceed 47 °C for up to 1 min [6].

Due to the importance of using proper irrigation on heat control during guided implantology, several studies have investigated the efficiency of different irrigation systems during guided osteotomies. Using chilled fluid and combined irrigation techniques, external and internal cooling systems have been evaluated in numerous in vitro studies [7,8] and animal studies [9]. The results of these studies indicated that the highest temperatures were noted when a conventional handpiece irrigator was used with a surgical guide, confirming that guided surgery interferes with proper irrigation during implant drilling. Subsequently, the possibility of thermal osteonecrosis could be higher in cases using conventional external irrigators when compared to internal ones [8,10,11].

Up to now, external irrigation is the most acceptable technique to avoid thermal injury during free hand implantology; however, the benefits of using this technique to minimize the heat in deeper osteotomy sites are controversial, leading researchers to find a solution to facilitate irrigation on entire osteotomy surface, including any drilling tissues covered with surgical guides. The incorporation of a cooling channel into a surgical guide has been suggested as an efficient technique to lead the fluid under the guide barrier during implant bed preparation [11,12]. Conversely, a recent study showed that adding a supplementary irrigation system to the surgical guide did not significantly reduce the temperature compared to conventional irrigation [13]. In this experiment, blockage of irrigation channels by bone debris was found to be a possible reason for reducing the efficiency of the proposed cooling system. The benefits of using capillary drills as an internal irrigation system to lead the fluid directly on the drilled bone surface are also controversial [14].

Considering all the proposed strategies to facilitate irrigation during guided implantology, the aim of this in vitro study is to improve the current design of surgical guides for implantology by incorporating a new irrigation system with an entry and exit channel into a 3D-printed surgical guide and evaluate its effect on heat generation during osteotomies.

## 2. Materials and Methods

Ethics approval was not required for this study; however, an animal ethics exemption was received from the animal ethics committee of Griffith University, as bovine ribs were used.

This was an in vitro study using fresh bovine ribs, as they have similar thermal conductivity and density as human bones [15]. After a power analysis study (85–90% power, significance at 0.05, detecting 2 °C difference with StDev of 1.5 °C), a sample size of 12 was considered ideal. All bone samples were sourced from a local butcher and all the residual soft tissues were cleaned off. Samples were marked by creating identification numbers carved into the bones using a rotary grinder (Ozito 170, Sydney, Australia) to facilitate identifying them after scanning.

### 2.1. Data Acquisition for 3D Planning

A pre-planning CBCT scan was obtained for all samples (Carestream CS 9300C, New York, NY, USA), set at 69 kV, 10 mA, and exposed for 11.30 s. The resolution was set to 180 voxels/mm^3^, allowing for 0.1 mm thick scans, and the field of view was 10 × 5 cm. A digital surface impression was also taken for all samples using an intraoral scanner (Trios 3, 3Shape A/S, Copenhagen, Denmark). The surface scan files were exported as standard tessellation language (STL) files. Then, the CBCT and the STL files were imported into the 3Shape Implant Studio software (3Shape A/S, Copenhagen Denmark) version 2018 1.2, a conventional commercial implant planning software for use in both clinical and laboratory environments.

### 2.2. Study Groups

To evaluate the changes in temperature observed with the new surgical guide designs, four groups were created:

Group A: irrigation built-in guide + exit channel (new design);

Group B: irrigation built-in guide (no exit channel);

Group C: external handpiece irrigation (conventional);

Group D: no irrigation (control group).

### 2.3. Surgical Guide Design and Manufacture

**Figure 1:**
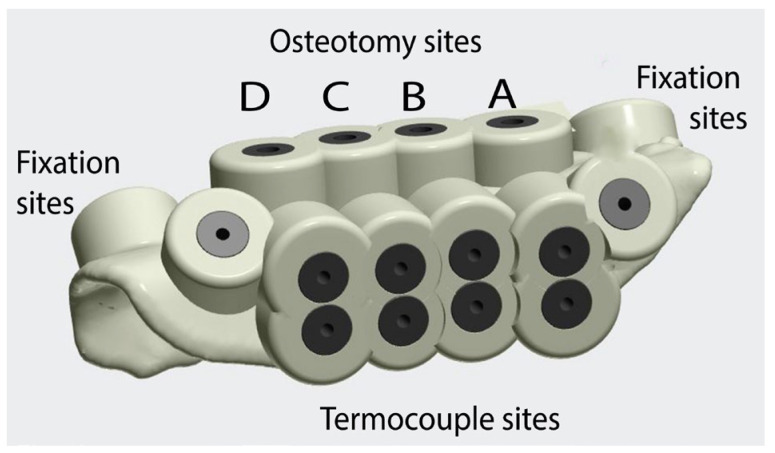
Surgical guide created for each sample, with groups A, B, C and D incorporated into the design. The osteotomy sites for each group are on the top of the guide, the fixation screw sites are at the edges of the guide, and the thermocouple sites perpendicular to the osteotomies, at 2 and 6 mm depths.

To ensure consistency at room temperature and allow for similar experimental conditions, we created one surgical guide for each sample, with the four groups built into the same surgical guide, mimicking one implant insertion per group (Figure 1). The digital planning for the guides was conducted using the 3Shape Implant Studio software (3Shape A/S, Copenhagen Denmark). This software allows the alignment of the two scans so accurate planning of implant or drilling site can take place.

**Figure 2:**
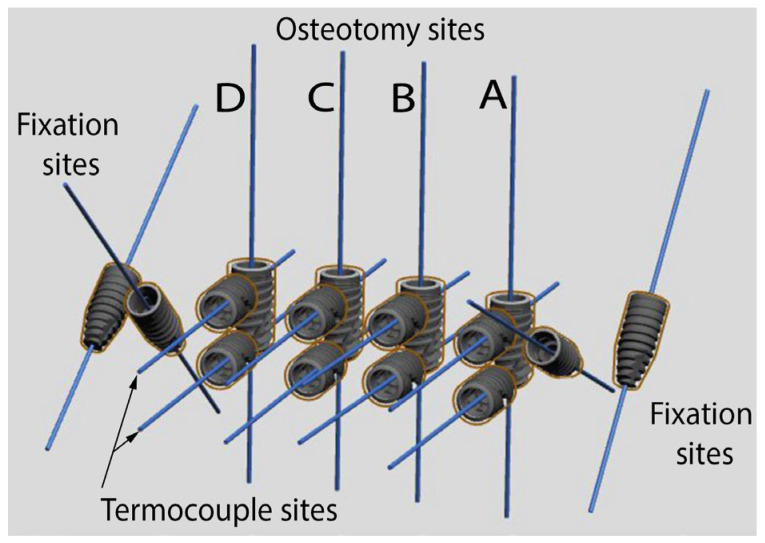
The digital planning of the guide was done using implant software (Implant Studio, 3 Shape A/S, Denmark). The osteotomy sites for the 4 groups, the fixation screws sites and the thermocouples insertion guides were made mimicking implants, and surgical sleeves were then incorporated into the guide. The varying osteotomy sites are indicated from A–D.

Four drilling sites were planned per guide (one for each group), with an inter-implant distance of 3 mm, all parallel to each other utilizing the software’s automated function (Figure 2). Titanium sleeves (Steco System Technik, Hamburg, Germany) with Ø 2.2 mm were added to guide the implant drill during the procedure. Each surgical guide was fixed to the bone sample with two fixation screws, and the holes for these were also incorporated into the guide design.

Two temperature measurement points were created into the guide, set perpendicular to the implant perforation at 2 mm depth (TC1) and 6 mm depth (TC2) by placing additional ‘phantom’ implants in this location to create a drilling hole (Figure 2). To precisely position these, insertion guides for the thermocouples were planned to reach a precise distance of 1 mm from the drilling surface. Sleeves of 1.3 mm diameter (Steco system technik, Hamburg, Germany) were incorporated into these insertion guides for the thermocouples. A surgical guide was then designed with a thickness of 2.2 mm with additional support structures added to allow the incorporation of irrigation channels. The surgical guide was then exported as a standard tessellation (STL) file (Figure 1).

**Figure 3:**
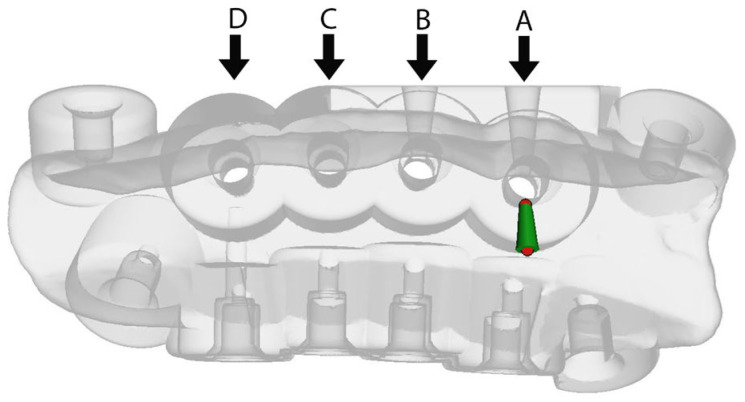
See-through illustration of the irrigation channel design showing all groups. These were designed in Meshmixer (Autodesk, San Francisco, CA, USA). Groups A and B had entry irrigation channels (near the arrows), and Group A had also an exit cooling channel (marked in green). Group C was used with a standard handpiece irrigation while group D had no irrigation.

To design the irrigation channels, the STL file of the surgical guide designs was imported into another computer software: Meshmixer (Autodesk, San Francisco, CA, USA). For group A (new design): two irrigation channels were incorporated into the surgical guide, so they emerged under the metal sleeve (Figure 3). The entry channel was used to insert the irrigation tubing, and the exit channel was used to vent the fluid and any debris to create a greater flow of coolant across the bur. For group B: only one entry irrigation channel was incorporated for the placement of the irrigation tubing. The channels were designed with a 3 mm diameter in a funnel shape, using the ‘add tube’ function, to place a 2.5 mm diameter irrigation tubing (Figure 3). The funnel shape allowed the tight and secure placement of the irrigation tubing. For groups C and D no irrigation channels were incorporated into the surgical guide.

The surgical guides were then additively manufactured in a surgical guide material (NextDent 5100, 3D Systems Inc., Rock Hill, SC, USA) and post-processed as per the manufacturer’s instructions with the metal sleeves being incorporated prior to post curing.

**Figure 4:**
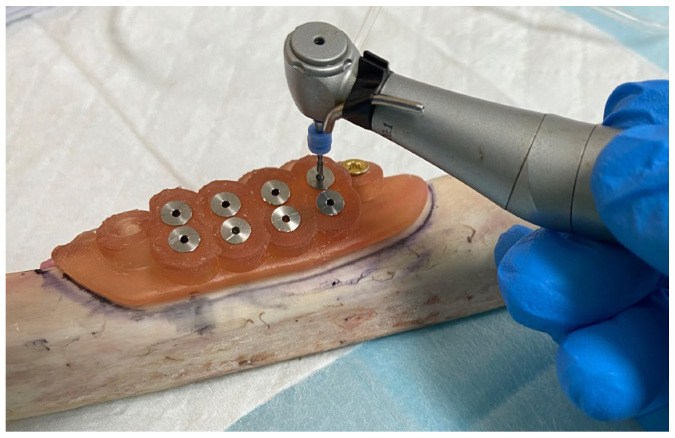
Preparation of the thermocouple channels using the ParaPost^®^ burs (Coltene, Cuyahoga Falls, OH, USA)with 1.14 mm diameter. The thermocouples had 1 mm in diameter and depth was measured individually in the software for each site, so it was at 1 mm of the osteotomy site.

On the day of the experiment, the specimens were allowed to reach room temperature, and all experiments were performed on the same day to ensure a standardized baseline for temperature measurements. The surgical guides were fixed to the bony specimens with two screws (Zenith, Mumbau, India) so they would not move throughout the experiment. The thermocouple insertion channels were drilled using ParaPost X Drills with a 4.5 size and a 1.14 mm diameter (Coltene, Cuyahoga Falls, OH, USA). The channel depth was measured individually in the software for each site, so it was at 1 mm of the implant drill. After cleaning the channels from any debris, the thermocouples (1 mm diameter) were inserted, and the holes were sealed and secured in place with an orthodontic wax (Figure 4).

**Figure 5:**
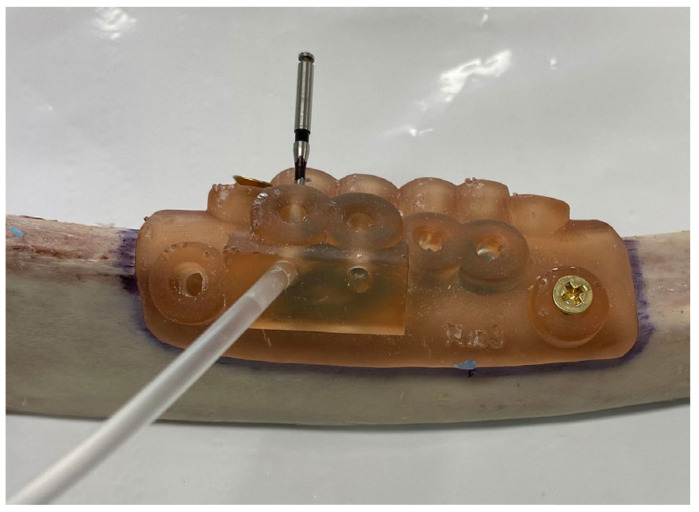
Surgical guide fixed in place with two fixation screws, showing the irrigation tube inserted into the channel (Group A). A drill was inserted into the other side to show where the exit channel is located.

The osteotomies for each group were then performed by using a 20:1 reduction contra angle (W&H, Bürmoos, Austria) with a pilot drill (Straumann Ø 2.2 mm, short) connected to an implant electric motor (Satelec, Viry-Châtillon, France), using the following setup: 800 rpm, torque at 45 Nm, irrigation fluid rate at 35 mL/min (Figure 5). For consistency, all osteotomies were performed by one calibrated operator (SP), to a depth of 8 mm. The drills were changed regularly as per the manufacturer’s instructions.

For Groups A and B the irrigation tubing was connected to the surgical guide, while for Group C it was connected directly to the handpiece (external irrigation), and for Group D the osteotomy was performed without irrigation (control).

**Figure 6:**
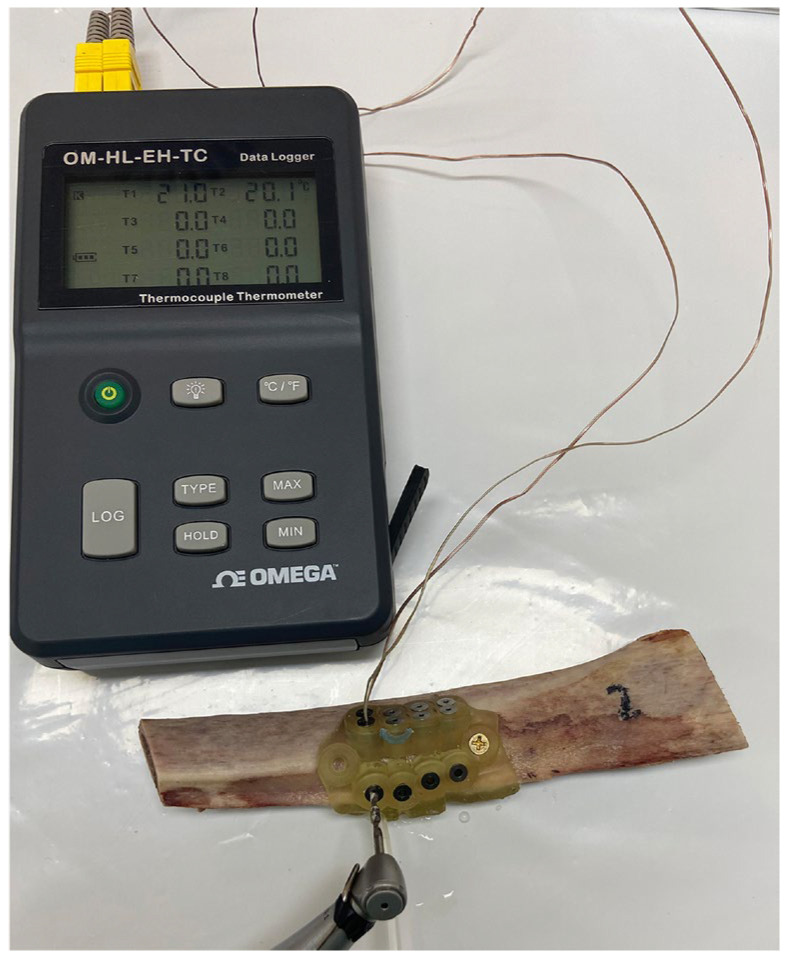
Implant osteotomy being performed with the irrigation entry and exit channel (Group A), measuring the temperature with thermocouples inserted into the thermocouple channels TC1 (2 mm) and TC2 (6 mm). These probes were coupled to an 8-channel data logger (OM-HL-EH-TC-K-CAL, OMEGA, Knoxville, TN, USA).

The temperature was recorded during each osteotomy using K-type thermocouples with a 1 mm diameter (TC Instruments, 5SRTC-TT-KI-36-0.5M, OMEGA, Knoxville, TN, USA). These probes were coupled to an 8-channel data logger (OM-HL-EH-TC-K-CAL, OMEGA, Knoxville, USA), as seen in Figure 6. The accuracy parameters were: ±0.8 °C ± 0.2%, and the reading time was for a minimum of 1 s sampling and 2 s logging intervals. All recorded data was transferred and stored on a personal computer using the OM-HL Logpro Software (OMEGA, Knoxville, TN, USA). The data was later downloaded in Excel format for statistical analysis. After each group was measured, the specimen was cleaned, dried, and the thermocouples were repositioned for the next group measurement.

### 2.4. Statistical Analysis

SPSS (version 27.0) was used for statistical analysis. Normality of the outcome variables, TC1 (2 mm depth temperature) and TC2 (6 mm depth temperature) were assessed using Shapiro–Wilk test. TC1 was not distributed normally. Median and interquartile ranges for presenting the data that were normally distributed, while mean and standard deviation were used for normally distributed data. Therefore, a non-parametric, Kruskal–Wallis test, was used to compare the TC1 temperatures between the four groups. Mann–Whitney U test was used to conduct pair-wise comparisons between the groups for TC1. One-way ANOVA was used to compare TC2 temperature between the groups and Games–Howell post hoc test was used to pair-wise comparison. A *p* value of <0.05 was considered significant.

## 3. Results

The highest temperatures measured among the three test groups (A, B and C) at 2 mm depth (TC1) and 6 mm depth (TC2) were 47.2 °C and 36.9 °C, respectively. Both were recorded in Group C (external irrigation), which was still less than the maximum temperature in Group D (control), with no irrigation (TC1: 51.4 °C, TC2: 52.6 °C), as shown in Table 1. There was a clear increase in the mean temperature from Group A to Group D (A < B < C < D), at both measurement points.

**Figure 7:**
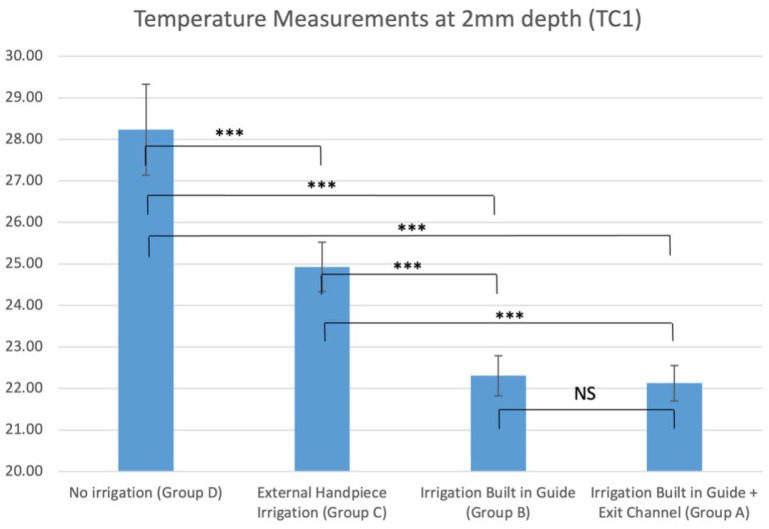
Average temperature measurements at 2 mm depth (TC1) in all groups (NS, non-significant, *** *p* < 0.001.

**Figure 8:**
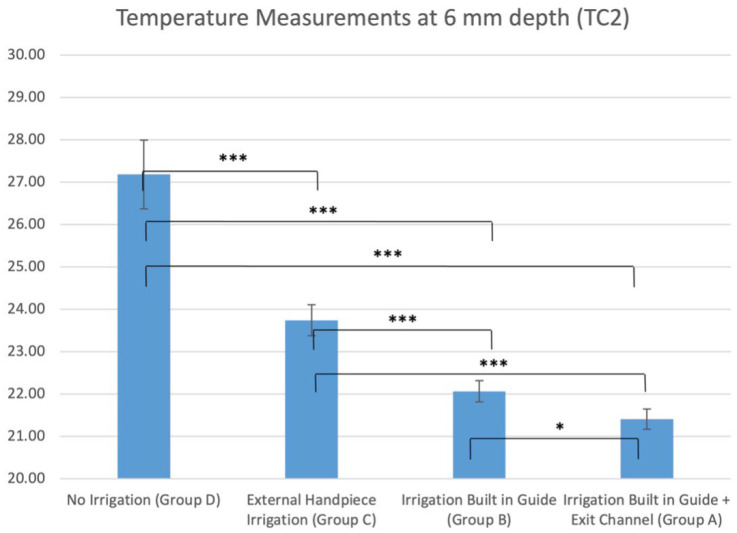
Average temperature measurements at 6 mm depth (TC2) in all groups (NS, non-significant, * *p* < 0.05, *** *p* < 0.001.

There were significant differences in recorded temperatures between all groups, both at 2 mm depth (Table 2 and Figure 7) and 6 mm depth (Table 2 and Figure 8). Group A (new guide) had highly significant lower temperatures (*p* < 0.001) compared to the control and external irrigation groups (mean temperature at TC1; 22.1 °C, TC2; 21.4 °C). Similar observations were noted in Group B, with significantly lower temperatures than Groups C and D (*p* < 0.01). When comparing Groups A and B, the new guide design showed better results, but these only reached significance in the TC2 measurement point.

The maximum temperature measured by TC1 was higher than TC2 for all groups; however, this difference did not reach significance (*p* > 0.05).

The mean deviation from baseline temperature in Group A with the new design never exceeds more than 2 °C. It should provide a concise and precise description of the experimental results, their interpretation, as well as the experimental conclusions that can be drawn.

## 4. Discussion

In the present study, surgical guides for implant placement were modified to incorporate a single cooling and a double cooling channel. The cooling channels were theorized to reduce heat generation and debris buildup on the bur. The results showed that a surgical guide with cooling channels incorporated improved irrigation fluid flow on the bur and into the osteotomy site. Furthermore, the addition of an exit channel has addressed the risk of obstruction with bone debris, resulting in even better temperature reduction. This finding supports the hypothesis that the addition of an irrigation channel into the surgical guide would minimize heat during the drilling of bone [11].

In the current experiment, the incorporation of one (Group A) or two (Group B) irrigation channels into the surgical guide have been shown to significantly reduce the temperature compared to external irrigation (Group C) or no irrigation (Group D). In the recent study by Stocchero et al. [13], the incorporation of the cooling channel did not make a statistically significant difference compared to the group with no irrigation or with a free hand technique without a surgical guide. In their study, a CBCT was taken of the surgical guides after the experiment with the obstruction of cooling channels being observed. A high-density drilling product was suggested as a possible cause for the insufficiency of coolant irrigation for this proposed surgical guide design. The cooling channels, in the current study, direct the cooling fluid directly onto the bur and around the metal sleeve, which without being cooled have significant overheating of bone [16]. This may also aid in reducing debris accumulated on the bur during an osteotomy [13]. Consequently, the integration of irrigation with this design has the potential to overcome one of the concerns related to guided osteotomy: heat-induced osteonecrosis of the bone [10,17]. The integration of an entry and exit irrigation channel may solve this problem as it would allow any debris to be vented from the bur during the osteotomy process.

In our study, the mean deviation from baseline temperature in Group A with the new design never exceed more than 2 °C, indicating that the surgical guide design would have the potential to keep the temperature within the safety zone in a clinical context during the cortectomy procedure. In most related articles, which investigated heat generation during osteotomies, the safety of the proposed irrigation systems was assessed based on the threshold value of 47 °C, which was first stated in 1984 by Eriksson, Albrektsson and Albrektsson [6]. Subsequently, if the temperature did not exceed 47 °C, the proposed system was considered safe for bone drilling, but it should be taken into consideration that in those studies, drilling performed on nonvital animal bone models and baseline temperature was room temperature (20–24 °C) at the time of the experiment, which was on average 15 °C lower than the baseline temperature on a human bone (37 °C) at the time of drilling [8,9,18]. Therefore, the amount of deviation from baseline temperature should be considered as the safety of any proposed irrigation system.

In the present study, the temperature value increased from Group A to Group D, with a limited increase from TC2 to TC1. This confirms that if the design of the irrigation system leads and maintains the fluid close to the osteotomy site and under the guide barrier, it is more likely to reduce the bone temperature during osteotomy. This finding is in accordance with the finding by Liu et al. (2016), who concluded that guided osteotomy using a drill with the internal hole was 3.6 times, and when using a cooling channel in the surgical guide was 1.95 times, more effective than conventional handpiece irrigation in heat control during osteotomy [10]. However, another study did not find any differences between internal and external irrigation systems in terms of heat generation during bone drilling [14]. In our study, the maximum temperature measured by TC1 was higher than TC2 for all groups, indicating that drilling of dense cortical bone with or without irrigation will generate more heat and highlights the need for effective irrigation to minimize thermal injury in dense cortical bone. This reinforces previous observations that the density of bone plays a more important role than the osteotomy depth in terms of heat generation during the drilling of the bone [7,13].

This in vitro study has limitations to a complete connection to a clinical context. The bones utilized were not living or had circulating blood which may have affected the outcome. Different structures in each bone may have also influenced the temperature readings. Future directions for this research could see this method conducted on a cadaver or live animal model to better simulate clinical conditions.

## 5. Conclusions

The proposed surgical guide with built-in entry and exit irrigation channels has significantly reduced heat generation during implant osteotomy compared to conventional external irrigation. The integration of an exit cooling channel has reduced heat and can resolve limitations related to the constant obstruction of irrigation channels found in previously designed surgical guides. This has an immediate practical application and can be incorporated into computer designing and 3D printing software.

## Figures and Tables

**Table 1:** Mean, standard deviation and maximum temperature recorded in all groups, at 2 mm depth (TC1) and 6 mm depth (TC2).

		A (Guide with Entry + Exit)	B (Guide with Entry Only)	C (External Handpiece)	D (Control)
TC1	Mean	22.1 °C	22.3 °C	24.9 °C	28.2 °C
(2 mm depth)	StDev	1.5 °C	1.7 °C	2.1 °C	3.8 °C
	Maximum	33.9 °C	45.9 °C	47.2 °C	51.4 °C
TC2	Mean	21.4 °C	22.1 °C	23.7 °C	27.2 °C
(6 mm depth)	StDev	0.8 °C	0.9 °C	1.3 °C	2.8 °C
	Maximum	28.9 °C	27.5 °C	36.9 °C	52.6 °C

**Table 2:** Pair-wise comparison of groups (the significance level is 0.05).

Groups in Comparison	TC1 (2 mm Depth)	TC2 (6 mm Depth)
A-B	0.3897	0.0352
A-C	0.0005	0.0000
A-D	0.0000	0.0000
B-C	0.0013	0.0005
B-D	0.0000	0.0000
C-D	0.0073	0.0004

## Data Availability

Data generated in this research project is available by contacting the last author of this paper via email. It is stored electronically as Excel worksheets.
